# No effect of intraspecific relatedness on public goods cooperation in a complex community

**DOI:** 10.1111/evo.13479

**Published:** 2018-04-29

**Authors:** Siobhán O'Brien, Elze Hesse, Adela Luján, David J. Hodgson, Andy Gardner, Angus Buckling

**Affiliations:** ^1^ Center for Adaptation to a Changing Environment (ACE) ETH Zürich 8092 Zürich Switzerland; ^2^ ESI & CEC, Biosciences University of Exeter TR10 9FE Cornwall United Kingdom; ^3^ CIQUIBIC, Departamento de Química Biológica, Facultad de Ciencias Químicas, CONICET Universidad Nacional de Córdoba Córdoba Argentina; ^4^ CEC University of Exeter TR10 9FE Cornwall United Kingdom; ^5^ School of Biology, Dyers Brae University of St Andrews Fife KY16 9TH St Andrews United Kingdom

**Keywords:** Cooperation, microbial communities, *Pseudomonas*, public goods, siderophores

## Abstract

Many organisms—notably microbes—are embedded within complex communities where cooperative behaviors in the form of excreted public goods can benefit other species. Under such circumstances, intraspecific interactions are likely to be less important in driving the evolution of cooperation. We first illustrate this idea with a simple theoretical model, showing that relatedness—the extent to which individuals with the same cooperative alleles interact with each other—has a reduced impact on the evolution of cooperation when public goods are shared between species. We test this empirically using strain of *Pseudomonas aeruginosa* that vary in their production of metal‐chelating siderophores in copper contaminated compost (an interspecific public good). We show that nonsiderophore producers grow poorly relative to producers under high relatedness, but this cost can be alleviated by the presence of the isogenic producer (low relatedness) and/or the compost microbial community. Hence, relatedness can become unimportant when public goods provide interspecific benefits.

The evolution of cooperation—any behavior that benefits another individual and is selectively favored, at least partly owing to that benefit (West et al. 2007)—is often affected by interspecific interactions (Ferrière et al. 2002; Harrison et al. 2008; Mitri et al. 2011; De Gasperin and Kilner 2015; Niehus et al. 2017). For example, the presence of other species may affect population structure of a focal species (Mitri et al. 2011) and increase competition for resources (Harrison et al. 2008) both of which can affect selection for cooperation. However, the greatest effects of interspecific interactions on the evolution of co‐operation are likely to occur when cooperative public goods benefit multiple species, such as toxin‐mediated killing and immune suppression of nematode hosts by *Photorhabdus* (ffrench‐Constant and Bowen 2000; Eleftherianos et al. 2007), as well as degradation of antibiotics (Lee et al. 2010), oil‐derived plastic polymers (Yoshida et al. 2016), wood (Zamocky et al. 2006), and cellulose (Zomorrodi and Segrè 2016) by microbial communities. Under these circumstances, there is likely to be selection for a decrease in the production—or even the complete loss—of public goods, as exemplified by the Black Queen hypothesis (Morris et al. 2012; Estrela et al. 2016), assuming species interact a significant amount of the time (Oliveira et al. 2014).

Cooperation is often favored if cooperators primarily interact with other cooperators (high relatedness), while it may be selected against if cheats come into contact with, and can exploit, cooperators (low relatedness) (Hamilton 1964). If, however, there are co‐operators everywhere—in the form of other members of the microbial community—whether interacting conspecifics are primarily cooperators or cheats may be trivial. Hence, when public goods are shared among species, the role of intraspecific relatedness in driving the evolution of the public good is likely to be less important. Here, we investigated how the presence of a natural compost community affects public goods cooperation in a focal species (siderophore production by the bacterium *Pseudomonas aeruginosa*) where the public good in this case provides both intra‐ and interspecific benefits. Bacteria, fungi, and plants produce a diverse range of siderophores (Hilder and Kong 2010).

The canonical function of siderophores is iron chelation: siderophores are released by cells in response to lack of iron, and the resulting iron‐siderophore complex is taken up by the siderophore producer or neighboring conspecific cells. Siderophores are costly to make, and hence nonproducing cheats can invade populations of co‐operating Pseudomonad species in well mixed (Griffin et al. 2004) and, to a lesser extent, structured in vitro environments (Kümmerli et al. 2009) and compost (Luján et al. 2015). Although siderophores can sometimes be pirated by other species (Meyer et al. 1999; Barber and Elde 2015; Galet et al. 2015), the high affinity between siderophore‐iron complexes and specific receptors makes them primarily an intraspecific public good in this context (Hohnadel and Meyer 1988; Buckling et al. 2007).

Siderophores can also act as public goods by remediating toxic levels of heavy metals (O'Brien et al. 2014; O'Brien and Buckling 2015). In this case, they bind to metals, preventing uptake by cells and rendering the metal (and hence the environment) nontoxic (Braud et al. 2009; O'Brien et al. 2014). Consequently, *P. aeruginosa* nonproducing cheats can outcompete siderophore producers in well‐mixed copper‐contaminated in vitro environments (O'Brien et al. 2014). Given that noniron siderophore‐metal complexes prevent metal uptake, siderophores can also be interspecific, as well as intraspecific public goods in this context. This was recently demonstrated in vitro, where *P. aeruginosa* cheat growth was increased by the addition of purified siderophores from different species (Hesse et al. 2018). However, it is unclear to which extent siderophores act as public goods in natural, metal‐contaminated environments: recent work reported ecological selection for increased siderophore production in contaminated soil and compost, suggesting fitness benefits of siderophores outweighed any cost associated with exploitation (Hesse et al. 2018).

Here, we investigate whether siderophores act as both inter‐ and intraspecific public goods in metal‐contaminated compost, by determining if a natural microbial community and siderophore‐producing conspecific bacteria can enhance the fitness of a nonsiderophore producing *P. aeruginosa* strain. Note that previous work has established that siderophores can act as metal decontaminating interspecific public goods in vitro (Hesse et al. 2018), but it is unclear if this is the case in natural environments such as compost or soil, where siderophores are known to be expressed (Marcschner and Crowley 1997). As a corollary of this prediction, being embedded within a microbial community will then reduce the importance of relatedness that is, the extent to which conspecific producers and nonproducers interact with each other—in determining selection for siderophore production in contaminated compost. After illustrating these predictions using a simple analytical model (Supplementary material 1), we test them empirically by measuring the growth of producer and nonproducer populations of *P. aeruginosa* in compost microcosms in a fully factorial design: alone or in competition with each other (high or low relatedness, respectively), in the presence and absence of toxic levels of copper, and in the presence and absence of the natural microbial community. We find that when public goods provide interspecific benefits, intraspecific relatedness can become relatively unimportant in determining the costs and benefits of cooperating.

## Methods

### BACTERIAL STRAINS

The *P. aeruginosa* strain PAO1 was used as a siderophore‐producing wild type, and an isogenic mutant strain PAO1*ΔPvdDΔPchEF* with both primary and secondary siderophores pyoverdine and pyochelin knocked out, was used as a siderophore negative mutant (Ghysels et al. 2004). Strains were modified by integrating a *lacZ* gene (with a gentamicin resistance cassette; Tn7‐gm‐*lacZ*), and a gentamicin resistance cassette (Tn7‐gm), at the *att*::Tn7 locus in PAO1 (PAO1 ^R^ *lacZ*) and PAO1*ΔPvdDΔPchEF (*PAO1*ΔPvdDΔPchEF^R^*), respectively (Choi and Schweizer 2006). *LacZ* gave PAO1 a blue pigment, so that it could be easily distinguished from PAO1*ΔPvdDΔPchEF* on Lysogeny Broth (LB) agar supplemented with 90 μg/mL 5‐Bromo‐4‐chloro‐3‐indolyl‐β‐Dgalactopyranoside (X‐gal). Resistance to gentamicin ensured both strains could be isolated from the natural community when plated on LB agar supplemented with 30 μg/mL gentamicin. To isolate the natural microbial community, 40 g of fresh 50% peat‐free compost (John Innes Potting Compost) was added to 200 mL M9 minimal salt solution and incubated shaken at 150 rpm at 28°C for 24 h. The supernatant was plated out neat on LB agar to verify the presence of a microbial community, and also on LB supplemented with 30 μg/mL gentamicin to verify the susceptibility of the community to this antibiotic.

### EXPERIMENTAL DESIGN

**Figure 1:**
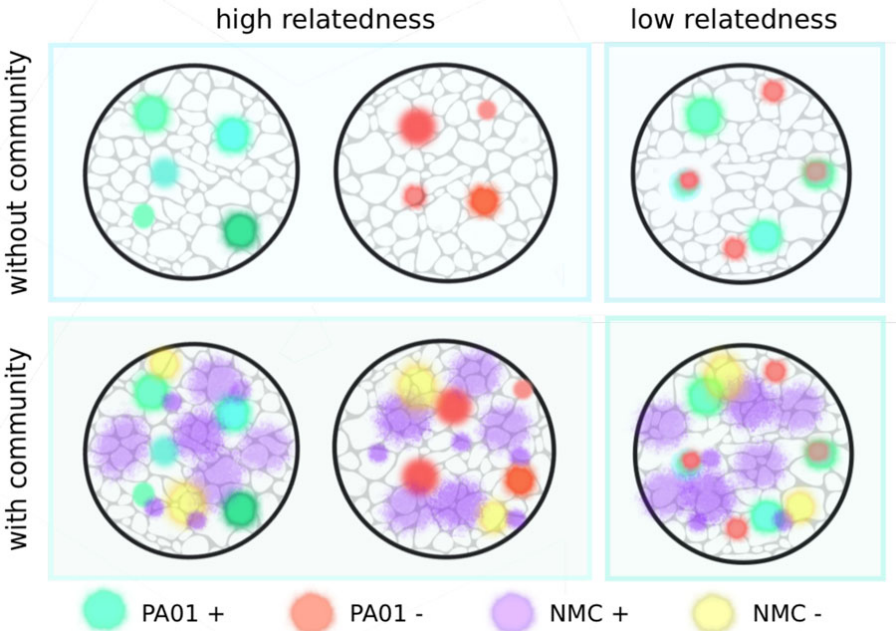
Experimental design for fully factorial experiment examining the effect of relatedness (*r*) and interspecific interactions on the relative fitness of nonproducers. Intraspecific relatedness was manipulated by inoculating single clones of producers (green) or nonproducers (red), alone (high *r*), or together (low *r*) (approach previously verified by Griffin et al. [2004]). Interspecific interactions were imposed by the addition of the natural compost community (NMC). The NMC is pictured as yellow or purple to illustrate the variation in siderophore production among members of the natural community (Hesse et al. 2018). This design was performed in the presence and absence of CuSO_4_. Six replicate microcosms for each treatment were established.

Compost microcosms were established with 30 g twice‐autoclaved 50% peat‐free compost in round 90 mm petri dishes. Compost microcosms, by definition, create structured populations, and hence maintain relatively high relatedness between interacting partners. To further manipulate relatedness in our microcosms, we inoculated either single clones (producer or nonproducer) separately, or both clones mixed together at 1:1 establishing conditions of very high and low(er) relatedness, respectively (as previously demonstrated in vitro by Griffin et al. [2004]; Fig. 1). To test whether the importance of intraspecific relatedness for cooperative behaviors is reduced in the presence of the community, we performed the following factorial experiment in 72 copper‐contaminated compost microcosms. Microcosms were inoculated with either ∼10^7^ colony forming units (CFU) of (i) PAO1 ^R^ *lacZ* (*n* = 24), (ii) PAO1*ΔPvdDΔPchEF^R^* (*n* = 24), or (iii) both strains in a 1:1 mixture (*n* = 24), so that the total inoculum was 10^7^ CFU's/mL in each case. Half of the microcosms were inoculated with 2 mL of the isolated community and 2 mL M9 minimal salt solution added to the remainder (Fig. 1). Microcosms were placed in an environmental chamber at 26°C and75% humidity. After 24 h we assessed population density of *P. aeruginosa* in each microcosm (see below) and to half of the microcosms in each treatment (*n* = 6), we added 2 mL filter‐sterilized 0.1 M CuSO_4_ or 2 mL ddH_2_O. Microcosms were returned to the environmental chamber at 26°C and 75% RH for five days, after which the density of PAO1 ^R^ *lacZ* and PAO1*ΔPvdDΔPchEF^R^* was assessed (see below).

### QUANTIFYING *P. aeruginosa* POPULATION DENSITY IN COMPOST

Initial density was quantified 24 h post inoculation (enabling bacteria to reach modest densities to withstand the addition of copper) and again five days after the addition of copper (final density). One gram of soil was transferred from each microcosm to 6 mL M9 minimal salt solution in 30 mL glass vials. Vials were shaken for 2 h at 28°C at 180 rpm, after which the supernatant was diluted in M9, and plated on LB agar plates supplemented with 90 μg/mL X‐gal and 30 μg/mL gentamicin. Plates were incubated at 37°C for 18 h, after which individual colonies could be identified and counted.

### STATISTICAL ANALYSES

We determined the population Malthusian growth rate (*m* per day) as ln(final density/start density)/day (Lenski et al. 1991) for producers and nonproducers in all populations. We were interested in how the different treatments affected the relative fitness of cheats (*W*), that is nonproducer growth rate/producer growth rate. For low relatedness treatments (where producers and nonproducers were directly paired), *W* was calculated as *m*(nonproducer)/*m*(producer) for each treatment combination. For high relatedness treatments, where there is no within‐microcosm pairing between producer and nonproducer populations, *W* was calculated using the mean growth rate of all six producer populations, that is *m*(nonproducer)/mean(*m*(producer) for each treatment combination. Given these different measures of relative fitness, we analyzed high and low relatedness treatments separately, using linear models with *W* as the response variable and the effect of copper and community (and 2‐way interaction) as factorial explanatory variables. To determine effects of relatedness, we analyzed the growth rates of producer and nonproducer populations in separate linear models, using growth rate (*m*) as the response variable, with Tukey Honest Significant Difference HSD posthoc tests. Copper toxicity was determined using the same fully factorial dataset, by directly comparing the growth (*m*) of producer and nonproducer in isolation in copper relative to their respective mean growth in iron. All data were analyzed using R version 2.15.1 (R Core Team 2012).

## Results

### INFLUENCE OF COPPER TOXICITY ON PRODUCER AND NONPRODUCER GROWTH RATES IN COMPOST MICROCOSMS

We assessed the toxicity of copper by comparing the growth rates of producers and nonproducers in copper‐contaminated compost, relative to their mean respective growth rates in the absence of copper. Consistent with previous studies, copper reduced growth of both strains (*t*‐test, alt = 1, producers (*t*_5_ = 6.88, *P* < 0.001, Fig. S1), nonproducers (*t*_5_ = 10.99, *P* = 0.0001, Fig. S1) but had a greater inhibitory effect on nonproducers than producers when grown in isolation (*t*_10_ = 1.96, *P* < 0.05, Fig. S1).

### THE MICROBIAL COMMUNITY ENHANCES NONPRODUCER FITNESS IN THE ABSENCE OF THE PRODUCER IN COPPER‐CONTAMINATED COMPOST

**Figure 2:**
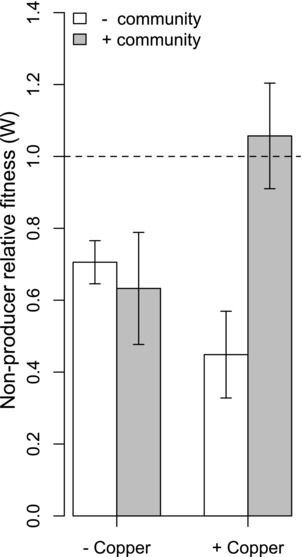
Nonproducer relative fitness (*W*) when producer and nonproducing strains are grown in isolation (high relatedness). *W* increases in the presence of the community, but only under copper‐contaminated conditions (community × copper interaction, *F*_1,20_= 7.25, *P *= 0.01), where siderophores act as interspecific public goods. When *W* = 1, growth of nonproducer and producer is equal. Error bars represent SE.

We first tested the hypothesis that the natural microbial community could increase the fitness of siderophore nonproducers relative to producers in copper‐contaminated compost, but to a lesser extent in noncontaminated compost. To this end, we focused solely on high relatedness treatments, to remove any confounding effects of direct interactions with conspecifics in the low relatedness treatments. As hypothesized, we found that nonproducer relative fitness was increased by the community, but only in copper‐contaminated communities, where siderophores can act as interspecific public goods (community × copper interaction, *F*_1,20_ = 7.25, *P* = 0.01, Fig. 2). In the absence of copper, the community had no effect on nonproducer relative fitness (Tukey HSD; *P* = 0.98). In copper, while there was a clear fitness benefit of siderophore production in the absence of the community (*W *< 1), the presence of the community enhanced nonproducer relative fitness, so that nonproducer and producer fitness did not differ (*W* = 1; Tukey HSD; *P* = 0.01, Fig. 2).

### RELATIVE FITNESS OF NONPRODUCERS IN A MIXTURE WITH PRODUCERS IS GREATER IN COPPER‐CONTAMINATED COMPOST

**Figure 3:**
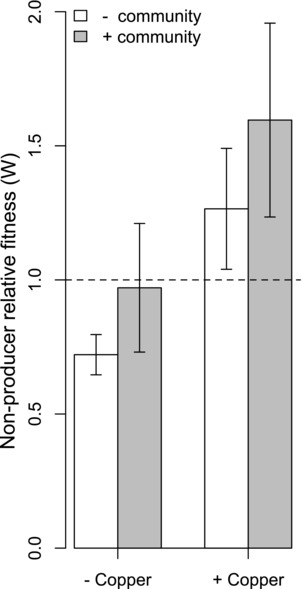
Nonproducer relative fitness (*W*) when producer and nonproducing strains are grown in coculture (low relatedness). *W* increases in copper contaminated environments, irrespective of whether the community is present (main effect of copper only: *F*_1,21_= 5.86, *P *< 0.05). This effect was independent of whether the community was present or absent (*F*_1,21_= 1.44, *P *= 0.24). When *W* = 1, growth of nonproducer and producer is equal. Error bars represent SE.

We next investigated if nonproducer fitness in the presence of producing conspecifics differed in copper‐contaminated (where siderophores act as interspecific public goods) versus copper‐uncontaminated soils, and whether this was contingent on the presence of the community. We found that the relative fitness of nonproducers in this context was greater in contaminated versus noncontaminated compost suggesting siderophores are a more exploitable public good in the context of remediation, than nutrient iron acquisition (main effect of copper only: *F*_1,21_ = 5.86, *P* < 0.05, Fig. 3). Moreover, this effect was independent of whether the community was present or absent (*F*_1,21_ = 1.44, *P* = 0.24, Fig. 3)

### THE COMMUNITY NEGATES THE EFFECT OF RELATEDNESS IN COPPER‐CONTAMINATED COMPOST

When siderophores act as an interspecific public good, our theoretical illustration (see supplementary material and Fig. S2) predicts that the role of intraspecific relatedness in shaping the costs and benefits of public good production will be reduced in the presence of the community. Comparing relative fitness between the high and low relatedness treatment is problematic because of the paired and unpaired nature of the low and high relatedness treatments, respectively. To avoid this, we separately analyzed growth rates of producers and nonproducers, in copper‐contaminated compost where community‐mediated public goods are important.

**Figure 4:**
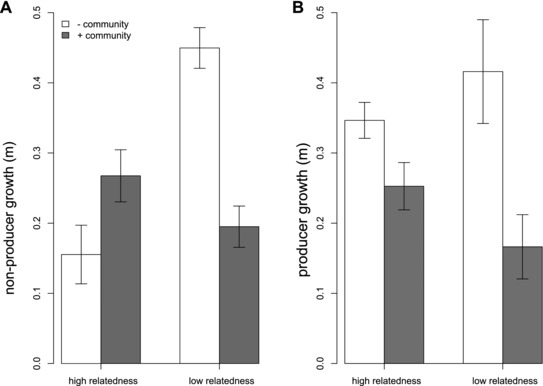
Malthusian growth rates for (A) nonproducers and (B) producers, grown in copper‐enriched compost. Nonproducer growth rate (a) was influenced by a strong interaction between relatedness and the presence of the natural community (community × relatedness interactions, *F*_1,20_ = 14.091, *P* < 0.0001), so that the community negated the effect of relatedness. Producer growth rate (b) was reduced by the presence of the community, irrespective of relatedness (main effect of community only: *F*_1,21_ = 11.73, *P *< 0.005). Error bars represent SE.

Nonproducer growth rate was influenced by a strong interaction between relatedness and the presence of the natural community (community × relatedness interaction, *F*_1,20_ = 14.09, *P* < 0.0001, Fig. 4A). Specifically, in presence of the community, no growth differences were observed between high and low relatedness treatments (Tukey HSD; *P* = 0.47), while in the absence of the community, low relatedness greatly increased the growth rate of nonproducers (Tukey HSD; *P* = 0.0001, Fig. 4A). By contrast, for producers, growth rate was entirely independent of relatedness (*F*_1,21_ = 0.03, *P* = 0.87, Fig. 4B), instead being simply reduced by the presence of the community (main effect of community only: *F*_1,21_ = 11.73, *P* = 0.003, Fig. 4B).

## Discussion

Here, we investigated how a natural compost community affects competition between *P. aeruginosa* public good (siderophore) wild‐type producers and nonproducers, under environmental conditions (copper contamination) where siderophores can act as both intra‐ and interspecific public goods. We found that, in copper‐contaminated compost microcosms, siderophores act as interspecific and intraspecific public goods, such that nonproducer fitness is increased to that of siderophore producers if either producers or the community are present.

We find that when the benefits of a public good are shared among community members, the importance of relatedness in determining selection of cooperative traits is attenuated. Our results have important implications for ecological theory, where theoretical and empirical studies using isolated species pinpoint to relatedness as being a key determinant of how selection acts on cooperative traits (Hamilton 1964; Griffin et al. 2004; West et al. 2007). Furthermore, our findings that microbial heavy metal detoxification is exploitable by nonproducers in compost has implications for siderophore‐mediated remediation, which relies on a constant supply of siderophore production by microbial communities.

Our finding that nonproducers incur a fitness cost even in nontoxic soils (i.e., *W* < 1), but that growth of both strains is equal in iron‐rich media (O'Brien et al. 2014), suggests that this soil is iron limited. Accordingly, bacterial siderophores are found in a wide variety of soils (Powell et al. 1980). However, unlike well‐mixed single species experiments (e.g., Griffin et al. 2004), we find the low fitness of nonproducers in compost cannot be completely rescued by conspecific producers. This is because in a spatially structured environment, the benefit of siderophores is most likely gained by other producers (Kümmerli et al. 2009, 2014). In contrast to our findings, Luján et al. (2015) found that the cost to *P. fluorescens* nonproducers in compost microcosms can be negated when growing in the presence of producers. This discrepancy may be explained by enhanced growth of *P. fluorescens* (carrying capacity (*K*) ∼10^6^) in soil relative to *P. aeruginosa* (*K* = ∼10^4^). Higher densities can increase relative fitness of nonproducers because nonproducers are better able to exploit the cooperative siderophore production of other cells when they are physically closer to them (Greig and Travisano 2004; Ross‐Gillespie et al. 2009).

Our conclusions have a number of caveats. First, our compost microcosms may differ with natural soil communities. However, to maintain common garden conditions and facilitate reproducibility, compost is an ideal medium to keep growth conditions constant. Second, we were not able to directly measure siderophore production by the community in situ, but have assumed that community siderophores are responsible for the tendency of the community to increase nonproducer fitness. However, we overcome this by using isogenic producer and nonproducer strains, so any effect of the community on nonproducer relative growth can be attributed specifically to siderophore production. Moreover, we have previously shown a direct interspecific benefit of five different siderophores in copper‐contaminated media (*P. aeruginosa* cheat fitness was enhanced relative to the wild type by the addition of siderophores; Hesse et al. 2018), and it is well established that microbial siderophores are abundant in soil under a wide range of pH levels (Powell 1980). Finally, our copper manipulations inevitably create further differences between the treatments over and above the effect of copper. Addition of copper sulphate marginally lowers pH (Hesse et al. 2018) potentially increasing iron solubility and reducing the need for siderophore production. However, this may have contributed to overall increased relative fitness of cheats in the presence of copper, it cannot explain the greatly reduced fitness of cheats when grown in isolation in copper‐contaminated compost. The addition of copper also increases siderophore production both physiologically (Teitzel et al. 2006; Braud et al. 2010) and as a result of ecological selection for higher siderophore producing taxa (Hesse et al. 2018). We therefore cannot rule out the possibility that the increase in fitness of cheats in copper is in part due to using other species’ siderophores to obtain iron.

The impact of community context on the fitness of a focal mutant is not limited to siderophores, and is likely to be relevant for a wealth of microbial “leaky” public goods, when the benefit cannot be solely directed to conspecifics. For example, *Escherichia coli* β–lactamases are public goods because they can benefit other species by inactivating ampicillin in the environment (Dugatkin et al. 2005). Furthermore, parasitic helminths have developed strategies that dampen their host's immune response (Allen and MacDonald 1998; Hartgers and Yazdanbakhsh 2006; Kamal and Khalifa 2006; Correale and Farez 2007; Cooke 2009; Hartmann et al. 2013), potentially indirectly diminishing the effect of the immune system on other pathogenic microbes. Interestingly, the dampening of the immune response initiated by these parasites has been demonstrated to increase virulence of viral infections such as human immunodeficiency virus (HIV), hepatitis B virus (HBV), and hepatitis C virus (HCV) (Kamal and Khalifa 2006).

Our findings lend some experimental support to the “Black Queen” hypothesis, which has recently examined the role of the community in mediating loss of functions acting as community‐wide public goods (Morris et al. 2012, 2014; Morris 2015). One species may benefit by losing an essential biosynthetic “leaky” function, provided the function is retained by at least one other species in the community. For example, in natural marine environments, most *Prochlorococcus sp*. lack the KatG enzyme while it is retained in *Synechococcus sp*. (Scanlan et al. 2009; Morris et al. 2011). Moreover, growth of unculturable bacteria can be enhanced by adding siderophores isolated from neighboring species, indicating that the community is driving the loss of costly biosynthetic traits (D'onofrio et al. 2010). In our study, the increase in relative fitness resulting from the loss of siderophore production in the presence of the community could be a first step toward the complete loss of production. However, it is notable that we found no actual fitness benefit to siderophore loss (relative to producers) in this case, suggesting that loss of key functions may instead be driven by drift rather than selection. Indeed, this view is consistent with work suggesting genome reduction is a consequence of drift and not selection (Kuo et al. 2009).

Here, we show that the cost of not making siderophores in heavy metal‐contaminated environments can be negated by the presence of a natural microbial community. However, this is in apparent contrast to recent work showing species that produce more siderophores are favored by ecological selection in comparable experimental settings (Hesse et al. 2018). This discrepancy cannot be wholly explained by greater assortment within—versus between—species, given that in this study nonproducers were rescued to the same extent by conspecifics and heterospecifics. Further work to explore these opposite responses to selection at genetic and ecological scales will be key to understand long‐term responses to shared public goods more generally and to explain the lack of effectiveness in using siderophore producing bacteria (as opposed to the direct addition of siderophores (e.g., Nair et al. 2007; Cao et al. 2012) to remediate metal‐contaminated environments (Wu et al. 2006; Kuffner et al. 2008; Li and Ramakrishna 2011; Rojas‐Tapias et al. 2012).

## CONFLICT OF INTEREST

The authors have declared no conflict of interest.

## DATA ARCHIVING

Data are available in supplementary material 2.

Associate Editor: S. Collins

Handling Editor: P. Tiffin

## Supporting information


**Fig. S1**. Copper reduced the growth rate (m) of both strains (T‐test, alt=1, producers (t_5_=6.8838, p<0.001, Fig S1), non‐producers (t_5_=10.987, p=0.0001, but had a greater inhibitory effect on non‐producers than producers when grown in isolation (t_10_ = 1.96, p < 0.05).
**Fig. S2**. The impact of relatedness (r) on the relative fitness of cheats (W) is expected to vanish in multispecies communities (lower *p*). Here we assume an average of *n* = 10 neighbors, siderophore cost *c* = 1.0 and siderophore benefit *b* = 0.2.

**Supplementary Material 1**. Theoretical model.
**Supplementary Material 2**. Raw data.
