# Supplementary material for: No effect of intraspecific relatedness on public goods cooperation in a complex community
Source: Evolution. 2018 Apr 29;72(5):1165–73. doi: 10.1111/evo.13479 (PMC5969229; doi:10.1111/evo.13479)
Supplement: Supplementary file 1 — Fig. S1. Copper reduced the growth rate (m) of both strains (T‐test, alt=1, producers (t5=6.8838, p<0.001, Fig S1), non‐producers (t5=10.987, p=0.0001, but had a greater inhibitory effect on non‐producers than producers when grown in isolation (t10 = 1.96, p < 0.05). Fig. S2. The impact of relatedness (r) on the relative fitness of cheats (W) is expected to vanish in multispecies communities (lower p). Here we assume an average of n = 10 neighbors, siderophore cost c = 1.0 and siderophore benefit b = 0.2. [file EVO-72-1165-s001.docx]

Supporting Information

Fig S1. Copper reduced the growth rate (m) of both strains (T-test, alt=1, producers (t_5_=6.8838, p<0.001, Fig S1), non-producers (t_5_=10.987, p=0.0001, Fig S1) but had a greater inhibitory effect on non-producers than producers when grown in isolation (t_10_ = 1.96, p < 0.05)

Fig. S2: The impact of relatedness (r) on the relative fitness of cheats (W) is expected to vanish in multispecies communities (lower *p*). Here we assume an average of *n* = 10 neighbours, siderophore cost *c* = 1.0 and siderophore benefit *b* = 0.2.
