# Supplementary material for: No effect of intraspecific relatedness on public goods cooperation in a complex community
Source: Evolution. 2018 Apr 29;72(5):1165–73. doi: 10.1111/evo.13479 (PMC5969229; doi:10.1111/evo.13479)
Supplement: Supplementary file 2 — Supplementary Material 1. Theoretical model. Supplementary Material 2. Raw data. [file EVO-72-1165-s002.docx]

Supplementary Material

*Theoretical Analysis*

Here, we provide a theoretical illustration of the investigated effect using a simple mathematical model. We consider that a focal cell has a Poisson-distributed number of neighbours, with mean *n*, with each neighbour being a conspecific with probability *p* and a heterospecific with probability 1-*p*, and each conspecific neighbour being a clonal relative with probability *r* and a nonrelative with probability 1-*r*. Each cell that invests in siderophore production suffers a direct fitness cost *c* and gives a fitness benefit *b* to each of the cells in its neighbourhood (including itself), in addition to a basic unit of fitness that we scale to unity. Assuming a vanishingly small incidence of cheating, a focal cooperator can expect to have *n* cooperative neighbours, giving it an expected fitness of *w*_coop_ = 1-*c*+(*n*+1)*b*. Conversely, a focal cheat can expect to have *n*(1-*pr*) cooperative neighbours, giving it an expected fitness of *w*_cheat_ = 1+*n*(1-*pr*)*b*. Accordingly, the relative expected fitness of cheats is given by *W* = *w*_cheat_/*w*_coop_ = (1+*n*(1-*pr*)*b*)/(1-*c*+(*n*+1)*b*). As ∂*W*/∂*r* = -*npb*/(1-*c*+(*n*+1)*b*) ≤ 0, the relative fitness of cheats is a monotonically decreasing function of relatedness, recovering classic insights (Hamilton 1964). And as ∂(∂*W*/∂*r*)/∂*p* = -*nb*/(1-*c*+(*n*+1)*b*) ≤ 0, this negative effect of relatedness is accentuated in the presence of conspecifics. Indeed, in the absence of conspecifics (*p* = 0), there is no effect of relatedness on the relative fitness of cheats (∂*W*/∂*r*|*_p_*_=0_ = 0). Moreover, the relative fitness of cheats is, in general, a monotonically increasing function of the frequency of heterospecifics (∂*W*/∂*q* ≥ 0, where *q* = 1-*p*; Fig. 2).
